# Antitumoral effect of local injection of TLR-9 agonist emulsified in Lipiodol with systemic anti-PD-1 in a murine model of colorectal carcinoma

**DOI:** 10.3389/fimmu.2023.1272246

**Published:** 2024-01-16

**Authors:** Anne-Laure Grindel, Nathalie Fretellier, Miguel Soares, Nabiha Bouzakher, Vincent Millot Maysounabe, Robin Santus, Olivia Bawa, Melody Wintrebert, Clémence Couquelet, Philippe Robert, Jean-Francois Emile, Claude Capron

**Affiliations:** ^1^ EA4340 Biomarqueurs en oncologie et onco-hématologie (BECCOH), Université Paris Saclay, Versailles, France; ^2^ Guerbet, Research and Innovation Division, Aulnay-sous-Bois, France; ^3^ INSERM US23 Analyse Moléculaire, Modélisation et Imagerie de la Maladie Cancéreuse (AMMICA), Villejuif, France; ^4^ Département d’anatomie Pathologique et de Cytologie, Hôpital Ambroise-Paré, Boulogne-Billancourt, France; ^5^ Immunology and Hematology Department, Hôpital Ambroise Paré, Boulogne-Billancourt, France

**Keywords:** immunotherapy, immunomodulation, lipiodol ^®^, preclinical model, colorectal carcimoma

## Abstract

**Introduction:**

Local treatments of cancer, including transarterial chemoembolization, could enhance responses to systemic immune checkpoint inhibitors such as anti-PD-1 antibodies. Lipiodol, a radiopaque oil, is widely used for transarterial chemoembolization as a tumor-targeting drug carrier and could be used in emulsion with immunomodulators. This study aimed at evaluating the antitumoral effect of intra-tumoral injection of Lipiodol-immunomodulator emulsions combined with systemic anti-PD-1 therapy in a murine model of colorectal carcinoma.

**Method:**

Mice (male BALB/c) with anti-PD-1-resistant subcutaneous CT26 tumors were injected with immunomodulators, emulsified or not with Lipiodol (N=10-12/group).

**Results:**

The TLR-9 agonist CpG displayed antitumor effects, while Poly I:C and QS21 did not. The Lipiodol-CpG emulsion appeared to be stable and maintained CpG within tumors for a longer time. Repeated intra-tumoral injections, combined with anti-PD-1, induced responses towards the tumor as well as to a distant metastatic-like nodule. This treatment was associated with an increase in proliferative CD8+ T cells and of IFN-γ expression, a decrease in proliferative regulatory T cells but also, surprisingly, an increase in myeloid derived suppressor cells.

**Conclusions:**

Local administration of CpG emulsified with Lipiodol led to an effective antitumoral effect when combined to systemic anti-PD-1 therapy. Lipiodol, apart from its radiopaque properties, is an efficient drug-delivery system. The formulated oil-in-water emulsion allows efficient loading and control release of CpG, which induces favorable immune modifications in this murine tumor model.

## Introduction

Immune checkpoint inhibitors (ICI), notably anti-PD-1 antibodies, have drastically changed the management of patients with cancer over the past decade. Indeed, ICI increase survival in several cancers including advanced melanoma and mismatch repair-deficient colorectal carcinoma (CRC) (1, 2). ICI can enhance antitumor immune responses of patients harboring “hot” tumors (3). By contrast, ICI seem ineffective in inducing antitumor immune responses in poorly immunogenic “cold” tumors. Thus, there remains a need for the development of new treatment combinations that would improve the rate of responses to ICI.

An emerging therapeutic strategy consists of combining ICI with locoregional therapies that release massive quantities of antigens following tumor cell destruction, thereby enhancing specific antitumor responses (4). Radiotherapy (5), thermal ablation (6), or transarterial chemoembolization (TACE) (7) have demonstrated synergistic effects with anti-PD-1 therapies. Phase III clinical trials are ongoing to evaluate the efficacy of such combinations, for example, the EMERALD-1 study comparing TACE alone to TACE with the anti-PD-L1 durvalumab (NCT03778957). Moreover, an abscopal effect (i.e. shrinking of tumors that were not the direct target of local therapy) has already been observed following combined treatment with radiotherapy and ICI (8), providing a strong rationale for combined local destruction and ICI.

Conventional TACE (cTACE) is a locoregional therapy used to treat primary liver cancer or liver metastatic colorectal carcinomas (CRC) (9, 10). Cytotoxic drugs are emulsified in Lipiodol (ethyl esters of iodinated fatty acids of poppy seed oil), a radiopaque liposoluble iodinated contrast agent used as a tumor-targeting vehicle, drug delivery system, and microvasculature embolizing agent. Lipiodol allows for a local and slow release of the chemotherapeutic agent into the tumor, leading to less systemic toxicity and increased drug concentration in tumoral liver nodules (11). Additionally, using Lipiodol as a carrier for immunostimulatory molecules such as OK-432, oligonucleotides (ODN) and siRNA can enhance antitumor responses (12–14).

Innate immune stimulators, including toll-like receptor (TLR) agonists or vaccinal adjuvants, can modulate the tumor micro-environment (TME) and therefore potentiate responses to anti-PD-1 therapies (15–17). Such immunomodulators are currently under investigation in solid tumors, notably injected locally in combination with systemic ICI (NCT04401995, NCT05638698).

We aimed to potentialize ICI-driven antitumor immune responses by combining a locoregional treatment with immunomodulators, emulsified in Lipiodol such as in a cTACE procedure, with an already approved systemic anti-PD-1 treatment. Three immunomodulators were selected for their antitumoral adjuvant properties observed in preclinical models and clinical trials: the TLR-3 agonist Poly I:C, TLR-9 agonist CpG-ODN, and saponin QS21. Activation of TLR-3 or TLR-9, both intracellular receptors, leads to improved antigen presentation following dendritic cell stimulation and antitumoral cytokines release (18). The mode of action of saponins as vaccine adjuvants is still poorly understood, but QS21 is thought to induce a Th1 response and enhance antigen capture by dendritic cells (19). It was hypothesized that local administration of an immunomodulator mixed with Lipiodol, used as a radiopaque carrier and tumor-targeting agent, could locally modulate the TME to promote anti-PD-1 antitumoral effects in both treated and distant non-treated nodules in an anti-PD1-resistant murine colorectal cancer model.

## Methods

### Mice and tumor induction

Healthy male BALB/c mice (6-8 weeks of age) were obtained from Janvier laboratories (Le Genest-Saint-Isle, France). All procedures, except for tumor measurements and anti-PD-1 injections, were performed under gaseous anesthesia (isoflurane). Euthanasia was performed when the tumors reached a maximum volume (details in **Supplementary Figure 1**) or became ulcerated.

The CT26 ATCC CRL-2638 colon adenocarcinoma cell line (ATCC, Manassas, VA, USA) was cultured as described previously (20). Cell concentrations for tumor induction are indicated in **Supplementary Figure 1**.

### Treatments

Treatment was initiated once the tumor reached 50 mm^3^. Poly I:C (Invivogen, San Diego, CA), QS21 (Euromedex, Souffelweyersheim, France) and CpG (Invivogen) were diluted in Xenetix 350 (iobitridol, Guerbet, Villepinte, France). Emulsions were prepared using Lipiodol Ultra Fluid (Guerbet). Mice (N=10-12/group) were randomly injected within tumors with either 50µg of Poly I:C, 20µg of QS21 or 40µg of class B CpG-ODN 1668 (or a control-ODN 2138). Anti-PD-1 (clone J43; BioXCell, Lebanon, NH, USA) was administered intraperitoneally twice a week (50µg/injection). Control mice tumors were injected with vehicle and did not receive anti-PD-1. Doses of each immunomodulator as well as anti-PD-1 were chosen based on previously published data (20–22).

Mice were considered responders to treatment when the treated tumor volume was reduced below the mean-2SD of that of controls, 8 days following the first i.t. injection.

### Follow-up

Tumor sizes were assessed at least three times per week by caliper measurement, and their volumes were determined using the formula: volume = (length x width²)/2. To study intra-tumoral (i.t.) Lipiodol deposition, tumors were imaged by 3D X-ray computed tomography (MicroCT, Quantum GX2, Perkin Elmer, Wellesley, MA) and by magnetic resonance imaging (MRI, Biospec 47/40, Bruker, Germany) with a T2 weighted sequence. Lipiodol quantification in the tumor rim was performed with the 3DSlicer software (V5 slicer.org) to colocalize MicroCT and MRI images. Tumor rims were defined, based on MRI images, as a region of 1mm width around the tumor edges. On the microCT images, a 2000 Hounsfield units threshold was applied for iodine detection in the tumor.

### Lipiodol emulsion characterization and *in vivo* biodistribution of CpG solutions

The stability of Lipiodol-CpG emulsions was assessed using the Turbiscan Tri-Lab (Formulaction, Toulouse, France). Four BALB/c mice bearing two subcutaneous CT26 tumors, one in each flank, were treated with an intra-tumoral injection of 40µg Cy5.5-coupled CpG alone (right tumor) or Lipiodol-Cy5.5-CpG emulsion (left tumor). Radiance was followed for 4 days in each tumor with IVIS Lumina III (Caliper Life Sciences, Hopkinton, MA, USA).

### Peripheral blood counts, plasma IFN-y quantification

Blood cell counts were determined using the MS4-S (Melet Schloesing, Osny, France). Plasma IFN-γ was quantified by ELISA according to the manufacturer’s instructions (Quantikine ELISA Mouse IFN-γ, R&D systems, Minneapolis, MN, USA).

### Flow cytometry

To study tumor-infiltrating leukocytes (TILs), tumors were cut into small pieces, incubated with a tumor dissociation kit, and dissociated with GentleMACS Octo Dissociator following the manufacturer’s instructions (Miltenyi Biotec, Bergisch Gladbach, Germany). Cell suspensions were then filtered, leukocytes were isolated by incubation with CD45 MicroBeads and sorted with MultiMACS following the manufacturer’s instructions (Miltenyi Biotec), before staining with antibodies. All antibodies used are described in **Table 1**.

**Table 1:** Antibodies and panels used for flow cytometry analysis.

Antibody (clone)	Species	Manufacturer
CD45 APC-Vio770 (REA737)	Human recombinant	Miltenyi
T cells markers		
CD3 FITC (REA641)	Human recombinant	Miltenyi
CD3 PE-Vio770 (REA641)	Human recombinant	Miltenyi
CD4 V450 (RM4-5)	Rat	BD Biosciences
CD4 Viogreen (REA604)	Human recombinant	Miltenyi
CD8 Viogreen (REA601)	Human recombinant	Miltenyi
CD8 BV786 (53-6.7)	Rat	BD Biosciences
CD44 PEVio770 (REA664)	Human recombinant	Miltenyi
CD62L PEVio615 (REA828)	Human recombinant	Miltenyi
B cells markers		
CD19 PEVio615 (REA749)	Human recombinant	Miltenyi
B220 Viogreen (REA755)	Human recombinant	Miltenyi
B220 PE-CF594 (RA3-6B2)	Rat	BD Biosciences
Exhausted markers		
PD1 PE (REA802)	Human recombinant	Miltenyi
PD-1 PercP-Vio700 (REA802)	Human recombinant	Miltenyi
PDL1 BV650 (MIH5)	Rat	BD Biosciences
PDL1 BV421 (MIH5)	Rat	BD Biosciences
TIM-3 APC (REA602)	Human recombinant	Miltenyi
NK cells markers		
CD49b FITC (DX5)	Rat	BD Biosciences
NKG2A PE (REA1161)	Human recombinant	Miltenyi
NKG2D BV711 (CX5)	Rat	BD Biosciences
NKp46 FITC (REA815)	Human recombinant	Miltenyi
NK1.1 APC (REA 1162)	Human recombinant	Miltenyi
Myeloid cells markers		
Gr1 PercP-Vio770 (RB6-8C5)	Human recombinant	Miltenyi
Gr1 PercP-Vio700	Human recombinant	Miltenyi
CD86 PE	Human recombinant	Miltenyi
CD206 AF 647 (MR5D3)	Rat	BD Biosciences
F4/80 FITC (REA126)	Human recombinant	Miltenyi
CD11c APC (REA754)	Human recombinant	Miltenyi
Ly-6C PE (REA796)	Human recombinant	Miltenyi
Ly-6G Vioblue (REA526)	Human recombinant	Miltenyi
CD11b VioGreen (REA592)	Human recombinant	Miltenyi
Cytokine markers		
TNFα AF 488 (MP6-XT22)	Rat	BD Biosciences
IFNγ AF 700, (XMG1.2)	Rat	BD Biosciences
IL-10 APC (REA1008)	Human recombinant	Miltenyi
Granzyme PE (REA226)	Human recombinant	Miltenyi
Treg cells and activation markers		
KI67FITC (REA183)	Human recombinant	Miltenyi
CD25 FITC (REA566)	Human recombinant	Miltenyi
MHC-II VioBlue (REA813)	Human recombinant	Miltenyi
CD69 PE (REA937)	Human recombinant	Miltenyi

Analysis of the spleen, draining lymph nodes and peripheral blood was also performed. Spleen and lymph nodes were dissociated with a potter. Red blood cells were lysed using PharmLyse (BD Biosciences, San Jose, CA). Immune cells from the spleen, draining lymph nodes and peripheral blood were then washed in phosphate buffered saline and stained with antibodies.

For Ki67 analysis, cell suspensions were fixed and permeabilized with a Fixation/Permeabilization kit (eBiosciences, San Diego, CA) before staining with antibodies.

For cytokine analyses, 1.10^6^ cells were incubated in 1mL of RPMI media 1640 (Sigma-Aldrich, Saint Louis, MO, USA) complemented with 10% fetal bovine serum and 2µL BD Golgi Plug Protein transport inhibitor containing brefeldin-A (BD Biosciences, San Jose, CA) for 4 hours at 37°C. Cells were then stained with surface antibodies, fixed and permeabilized with a Fixation/Permeabilization kit (BD Biosciences) before incubation with anti-cytokine antibodies.

Flow cytometry acquisitions were performed on a LSR Fortessa X20 (BD Biosciences). Data were blindly analyzed with DIVA and FlowJo software (BD Biosciences).

The unsupervised approach for the identification of differences between groups of mice was performed using multiparametric flow cytometry analysis GPU accelerated t-distributed stochastic neighbor embedding (t-SNE-CUDA).

Flow cytometry samples from 10 mice were manually gated on CD3+ T lymphocytes among CD45+ cells. The CD3+ T cells were down-sampled to 2500 cells per sample, t-SNE-CUDA were generated by concatenation of 2500 CD3+ cells per sample with five control mice and five mice having received the combination treatment. Total concatenated cells where the merged into a single expression matrix prior to t-SNE analysis. CD4, CD8, CD25 and Ki67 parameters were used to calculate the t-SNE-CUDA. Clusters were assessed for FlowSOM (flow cytometry data analysis using self-organizing maps) clustering method. Clustering and t-SNE CUDA maps were performed using OMIQ software (OMIQ, Inc., Santa Clara, CA, USA).

### Immunohistochemistry

Freshly collected half-tumors were placed in paraformaldehyde for 24 h at room temperature. After fixation, the dehydrated tumors were embedded in paraffin. Sections of 4µm thickness were cut. Hematoxylin-eosin and CD3 staining were performed on whole slides. Double staining was performed on tissue array sections containing two 0.6mm large cores of each tumor. All antibodies used are described in **Supplementary Table 1**. All double staining was performed with the Discovery Ultra (Roche Diagnostic/Ventana, Illkirch, France), and data were analyzed blindly using ImageJ (National Institutes of Health).

### Statistical analysis

GraphPad Prism software (9.5) was used to analyze data and determine statistically significant differences between groups.

The Shapiro-Wilk test was used to assess the normality of the data distribution. Data showing non-homogeneous variances or a non-normal distribution were analyzed using the Kruskal-Wallis test followed by Dunn’s test when the Kruskal-Wallis test was found to be significant. When comparing only two groups, an unpaired t-test or Mann-Whitney test was performed when the data showed a normal or non-normal distribution, respectively. The log-rank test was used to compare survival probabilities. Statistical significance was set at p <0.05.

## Results

### Antitumor effects of Lipiodol-CpG emulsions, especially when combined with systemic anti-PD-1 injections

**Figure 1:**
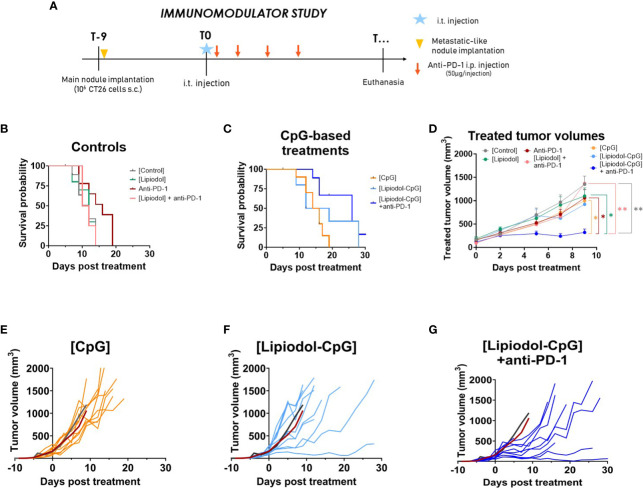
The TLR-9 agonist CpG induces an antitumor response alone, when emulsified in Lipiodol and when combined with systemic anti-PD-1. **(A)** Study design. Kaplan-Meyer pseudo-survival curves following intra-tumor injection of **(B)** control treatments or **(C)** TLR-9 agonist CpG (N=10 mice/group). Log-rank test was performed. Compared to the control, a significant increase in pseudo-survival is observed for CpG alone (p<0.01), Lipiodol-CpG (p<0.05) and the combination group (p<0.001). Combination group also present a significant increase in pseudo-survival compared to the anti-PD-1 alone group (p<0.05). **(D)** Mean treated-tumor volumes after intra-tumor injection. Data are presented as mean ± SEM, a two-way ANOVA was performed, * p<0.05, ** p<0.01. **(E)** Individual treated-tumor volumes of mice injected with CpG alone, **(F)** Lipiodol-CpG, **(G)** Lipiodol-CpG and systemic anti-PD-1 injections. In **(E-G)** dark and red curves represent the mean of tumor volumes of controls or anti-PD-1 treated animals, respectively.

The antitumoral effects of the three immunomodulators tested were investigated first (**Figure 1A**). When compared to controls (**Figure 1B**), Poly I:C and QS21 did not appear to increase pseudo-survival regardless of the form or combination used (**Supplementary Figures 2A-C**), whereas CpG-based treatments led to better outcomes (**Figure 1C**). Due to early tumor ulceration, some mice had to be euthanized before reaching the maximal volume (4 in the control group, 3 in the Lipiodol-CpG + anti-PD-1 (combination group), 2 in the anti-PD-1, Lipiodol and Lipiodol-CpG groups, and 1 in the CpG alone group). A significant increase in pseudo-survival was observed for CpG treated mice compared to the control group (p<0.05), and for mice of the combination group compared to both the control group (p<0.001) and the anti-PD-1 group (p<0.05). A significant slowdown in tumor growth was observed in the combination group compared to other groups (**Figure 1D**). All responsive mice belonged to the Lipiodol-CpG group (1/10 mice) or combination group (2/10 mice) (**Figures 1E–G**). Interestingly, compared to the mean of the control group (dark curve) and the mean of the anti-PD-1 group (red curve), 3/10 mice in the Lipiodol-CpG and 10/10 mice in the combination group have a slower treated tumor growth (**Figures 1F, G**), contrary to the CpG group where all mice have a similar tumor growth to the mean of the two control groups (**Figure 1E**). No antitumoral effect of anti-PD-1 alone or Lipiodol + anti-PD-1 treatment has been observed in this model.

### Lipiodol as a radiopaque drug delivery system increases CpG tumor retention

**Figure 2:**
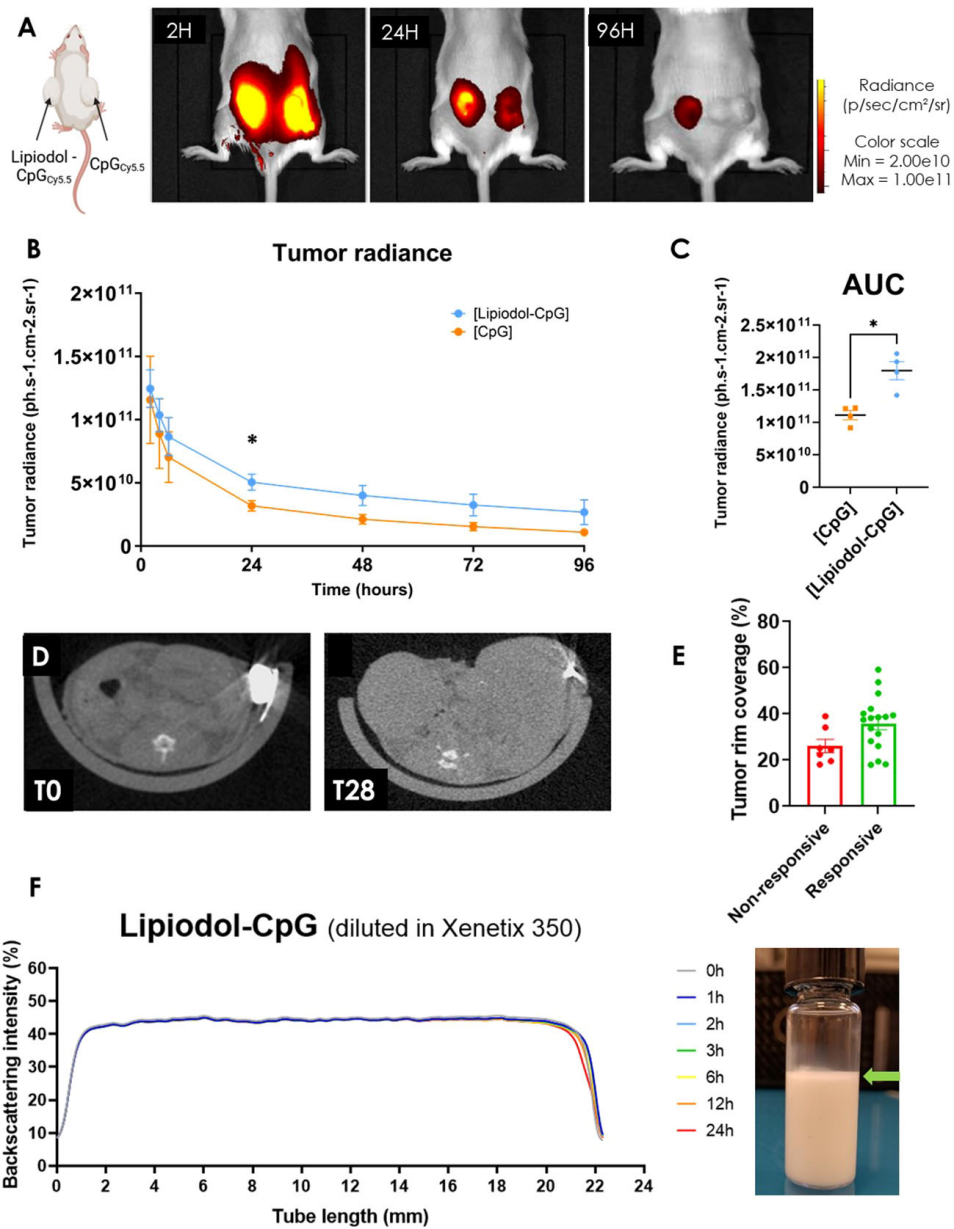
Lipiodol emulsion allows increased retention and longer delivery of CpG in the tumor. **(A)** *In vivo* fluorescence imaging of a mouse treated with an i.t. injection of either Lipiodol-CpG emulsion (left tumor) or CpG (right tumor), 2, 24 and 96 hours following the injections (N=4 mice). **(B)** Tumor radiance and **(C)** corresponding area under the curve of both tumors was determined. A Mann-Whitney test was performed. **(D)** MicroCT imaging of a treated tumor injected with Lipiodol-CpG emulsion immediately following the i.t injection and 28 days after the injection. **(E)** Iodinated deposition at the tumor rim is more important in responsive mice. **(F)** Backscattering intensity measured by Turbiscan of a Lipiodol-CpG diluted in Xenetix^®^ 350 emulsion, called “Lipiodol-CpG” in the *in vivo* studies. Very small clarification is observed at the top of the tube 24 hours after the emulsification process of Lipiodol-CpG emulsion, demonstrating stability (green arrow). Data presented as mean ± SEM, * p<0.05.

Mice developing two similar CT26 tumors were treated with CpG conjugated with Cyanine 5.5 either alone (right tumor) or emulsified in Lipiodol (left tumor). A significantly slower decrease in the radiance of the tumor treated with Lipiodol-CpG was observed, with a higher half-life than that of CpG alone (69.2 ± 11.5 versus 43.2 ± 1.7h, p<0.05) (**Figures 2A, B**). This resulted in a 1.6 -fold higher global 96h-exposition to treatment (**Figure 2C**). Lipiodol deposition as a biomarker of response was also assessed by microCT (**Figures 2D, E**). Interestingly, the radio-opacity of Lipiodol was still detectable within tumors by microCT 28 days after a single emulsion injection. The total iodine quantity was similar, yet tumor rim coverage tended to be associated with the tumor response. This prolonged presence of Lipiodol demonstrated by the microCT at 28 days, and the increased biodisponibility of CpG emulsified in Lipiodol (*in vivo* fluorescence imaging for 4 days) could be explained by the high stability of the Lipiodol-CpG emulsions (**Figure 2F**). Lipiodol-CpG emulsions were direct (oil-in-water), without any coalescence observed macroscopically or microscopically immediately after the emulsification. The Turbiscan analysis demonstrated a very small clarification process (**Figure 2F**, **Supplementary Figure 3**).

### Effects of repeated intra-tumoral injections

**Figure 3:**
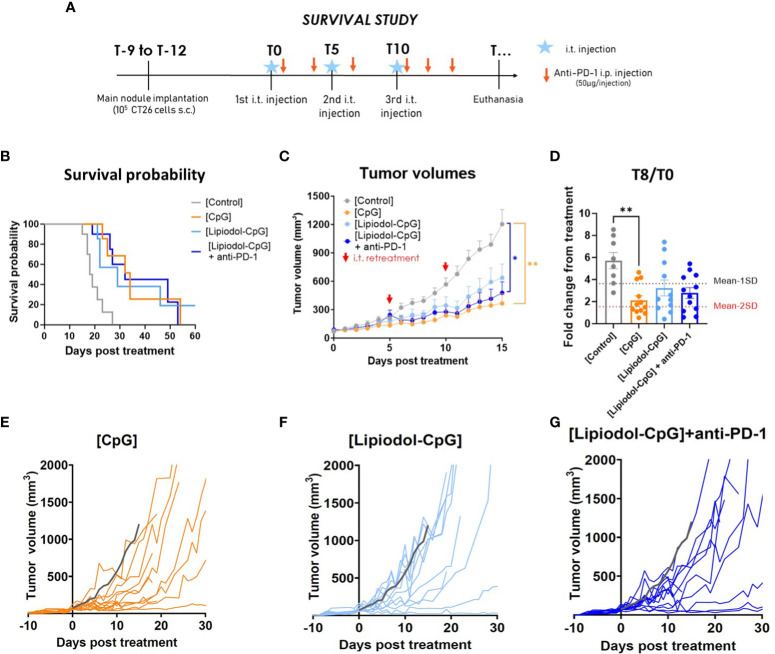
Repeated treatments with CpG, alone or emulsified and combined with anti-PD-1 led to increased antitumoral effect in a single tumor model. **(A)** Study design. **(B)** Kaplan-Meyer pseudo-survival curves following the first i.t. injection (N=12 mice/group). A log-rank test was performed. **(C)** Mean treated tumor volumes following the first i.t. injection. Retreatments were performed by i.t. injections at T5 and T10 (red arrows). A two-way ANOVA was performed. **(D)** Fold change in treated-tumor volume at T8. Individual treated-tumor volumes following the first i.t. injection of **(E)** CpG, of **(F)** Lipiodol-CpG and **(G)** of Lipiodol-CpG combined with systemic anti-PD-1 injections. Grey curves represent the mean of the control group. As Kruskall-Wallis was found positive, a Dunn’s post-test was performed. Data presented as mean ± SEM and individually, * p<0.05, ** p<0.01, *** p<0.001.

To avoid a too rapid tumor evolution in the control group, a second study was performed by injecting only 10^5^ cells, resulting in a less severe model (0% of death at T10 for control mice at 10^5^ versus 40% with 10^6^ cells). Under these new conditions, better control of tumor growth was achieved using repeated i.t. injections (**Figure 3A**). Only 5, 3 and 3 of the 12 mice had to be euthanized before reaching maximal volume due to tumor ulceration in the CpG, Lipiodol-CpG and combination groups, respectively. Surprisingly, repeated treatments with CpG alone or Lipiodol-CpG induced a similar increase in pseudo-survival and a significant slowdown in the treated tumor growth as in the combination group (**Figures 3B, C**). In this protocol, 5/12 mice responded to CpG, 4/12 to Lipiodol-CpG and 4/12 to Lipiodol-CpG + anti-PD-1 (**Figure 3D**).

A complementary experiment was carried out comparing effects of CpG-ODN with a control-ODN to investigate whether observed effects with CpG on CT26 tumor are dependent on TLR9 involvement (**Supplementary Figure 4**). No effect was observed after i.t. administration of control-ODN while increase in survival probability and decrease in tumor burden were observed following i.t. administrations of CpG.

### Abscopal effects of the treatment and local immune activation of CD8+ T cells

To determine whether treatment effects were due to an enhancement of the immune response, the presence of an abscopal effect was investigated and immune cell populations were analyzed within tumors, secondary lymphoid organs, and blood.

**Figure 4:**
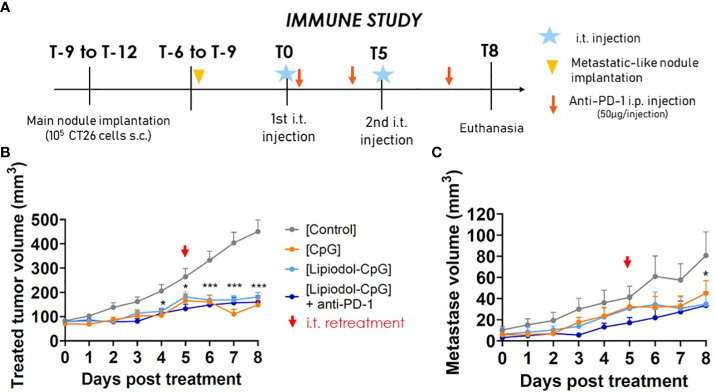
Abscopal effects of the treatments. **(A)** Study design. **(B)** Treated-tumor and **(C)** untreated metachronous metastatic-like tumor volumes following the first i.t. injection (N=12 mice/group). Two-way ANOVA were performed, *p < 0.05, ** p < 0.01.

Immune characterization was conducted in a separate experiment than the survival study, so that organs of interest could be sampled at the same time for all mice, few days after the last treatment, at a delay where tumors start to respond to the treatments. This study was continued with a less aggressive design of metachronous metastasis (**Figure 4A**). In terms of main tumor growth and responsive profiles, results were similar (**Figure 4B**) as those shown in **Figure 3**. Interestingly, a significant decrease in the metastatic nodule growth was observed for all treated groups, disclosing an abscopal effect (**Figure 4C**): 9/12 mice in the CpG group, 7/12 in the Lipiodol-CpG group and 10/12 in the combination group. As no specific pattern in cells proportions either in flow cytometry or IHC has been observed for responsive mice, analyses were performed by comparing treatments, regardless of the response.

Mice treated with CpG had significant splenomegaly, with spleens weighing twice as much as those of control mice (**Supplementary Figure 5A**). Blood analysis showed a significant decrease in red cell counts in the CpG and Lipiodol-CpG groups compared to the controls (p<0.05). Only minor differences were observed in the platelet and leukocyte counts between the groups (**Supplementary Figures 5B–E**).

**Figure 5:**
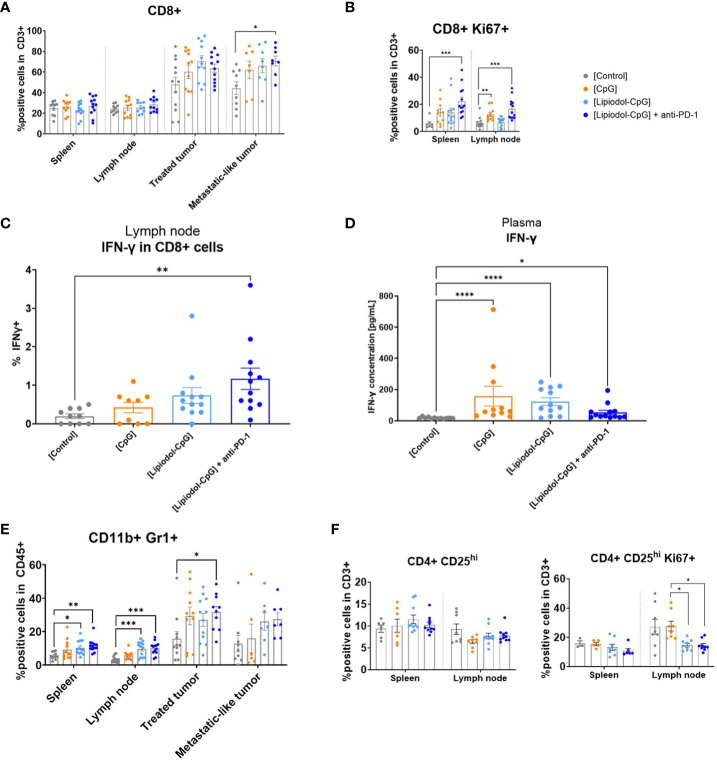
Analysis of tissue-infiltrating CD8+ lymphocytes, IFN-γ expression and proportions of suppressive cells. At T8, spleen, draining lymph nodes and both tumors were harvested (N=12 mice/group). Half of the treated tumors were used for flow cytometry analysis. The tissues sampled were dissociated before labelling. Inoperable samples led to a few missing data. **(A)** Proportion of CD8+ cells among the CD3+ population in the spleen, draining lymph nodes, treated tumor and metastatic-like one. **(B)** Proportion of CD8+ Ki67+ cells in the CD3+ population. **(C)** Secretion of IFN-γ by non-stimulated CD8+ from lymph nodes **(D)** IFN-γ concentration in plasma measured by ELISA. **(E)** CD11b+ Gr1+ MDSC cells among the CD45+ population in all tissues. **(F)** Proportion of CD4+ CD25^hi^ Tregs among CD3+ cells and of CD4+ CD25^hi^ Ki67+ among CD3+ cells in the spleen and lymph nodes. As Kruskall-Wallis were found positives, Dunn’s post-tests were performed. Data presented as mean ± SEM and individually, *p<0.05, **p<0.01, ***p<0.001, ****p<0.0001.

Although there was an increase in CD3+ cells in tumors (**Supplementary Figure 6**), no difference in CD4+ cells was observed within the tumors or lymphoid organs. The proportion of CD8+ T-cells within the CD3+ population increased in treated and metastatic nodules, with a significant difference between the control and the combination groups within the metastatic nodule (**Figure 5A**). Interestingly, although no difference in the proportion of CD8+ cells was observed in the spleen and lymph nodes, proliferating Ki67+ CD8+ lymphocytes increased in all treated groups, especially in the combination group (**Figure 5B**). No difference was observed in Natural killer (NK), B cells, macrophages or dendritic cells in all organs by flow cytometry (data not shown).

A significant increase in CD8+ and CD4+ T cells expressing IFN-γ was observed in the lymph node of the combination group (**Figure 5C**, **Supplementary Figure 7**). No difference was observed for TNF-α, IL-10, and granzyme B expression (data not shown). Analysis of plasma IFN-γ by ELISA also demonstrated increased secretion of this cytokine in all treated groups compared to controls (**Figure 5D**).

### Combined treatment modulates local and circulating immunosuppressive cells

Myeloid-derived suppressor cells (MDSC) defined as CD11b+ Gr1+ in CD45+ cells are immature myeloid cells with immunosuppressive properties (23). Surprisingly, MDSC were found in greater proportions in the combination group than in the controls, in the spleen, draining lymph nodes, and locally in the treated tumor (**Figure 5E**). Moreover, a non-significant decrease of the immunosuppressive CD4+ CD25^hi^ Treg population was observed in all treated groups compared to the controls in the lymph nodes, by flow cytometry (**Figure 5F**). CD4+ CD25^hi^ Ki67+ proliferating cells were also in significantly less proportions in the draining lymph nodes from treated tumors (**Figure 5F**).

### Unsupervised analysis of T cells and Tregs in the lymph nodes

Next, a multiparametric flow cytometry analysis was performed to characterize CD3+ T cells in lymph nodes from mice. A t-stochastic neighbor embedding (t-SNE) CUDA analysis that reduces all data to 2 dimensions using the concatenated data from lymph node of 10 mice was conducted.

**Figure 6:**
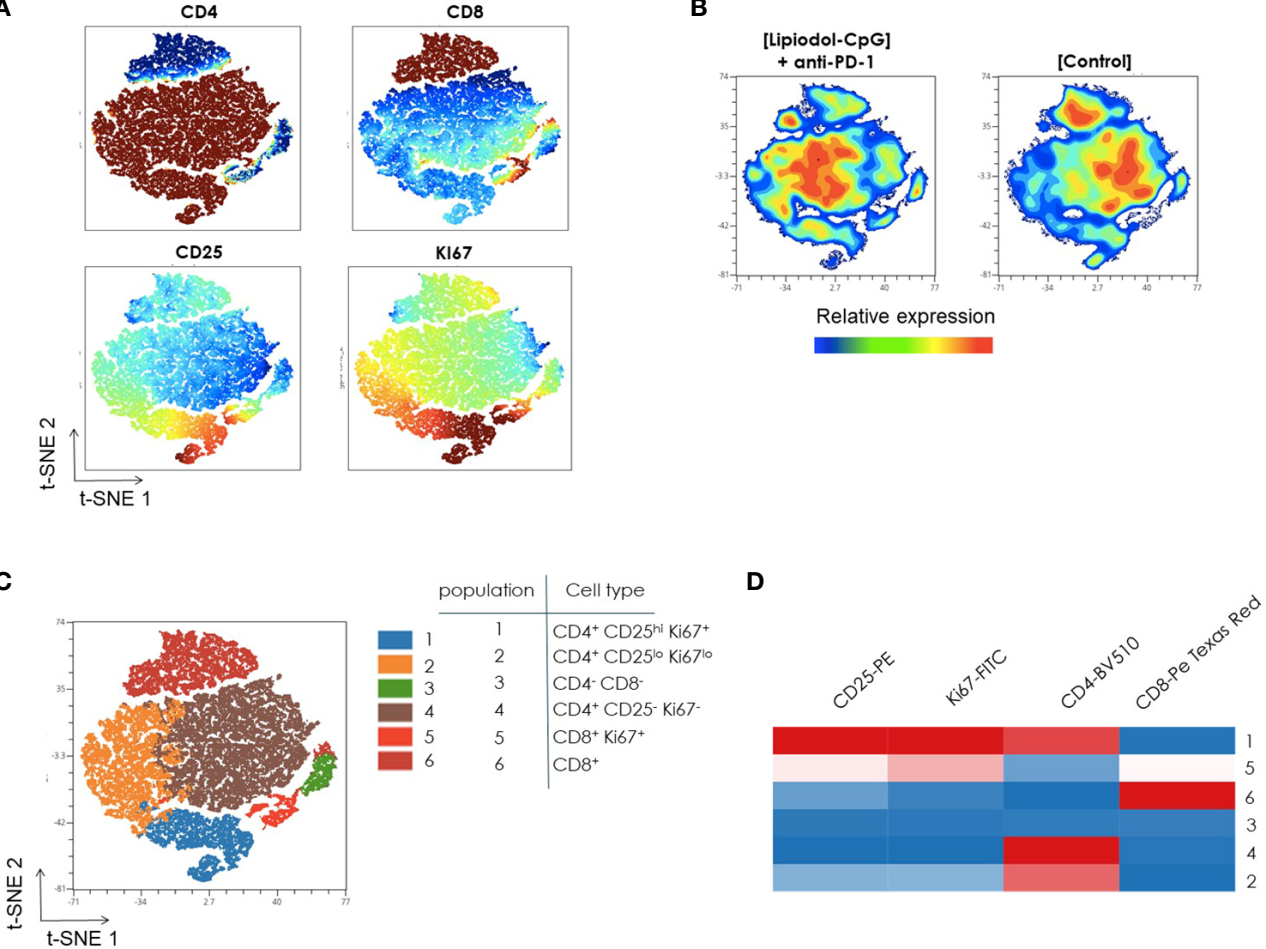
Unsupervised flow cytometric analysis of T-cells from lymph nodes. CD3+ Lymphocyte populations were concatenated from lymph node samples after downsampling (five donors from each sample type) and analyzed with t-SNE (t-stochastic neighbor embedding). **(A)** Identification of cell subsets using t-SNE. Shown here is the expression pattern of each individual marker in lymph node after gating on CD3+ cells. Color code from dark blue to dark brown indicates increased expression level of each marker. **(B)** A t-SNE-CUDA map displays CD3+ T cell populations in control and treated mice. Colors represent expression levels (blue: low, green: intermediate, red: high). **(C)** FlowSOM-based meta clusters were overlaid onto the t-SNE map as a color dimension to identify the major cell subsets in CD3+ T cells from lymph node. The selection of clustering parameters involved the application of FlowSOM with a grid of 7 x-maps and 7 y-maps. The determination of the number of metaclusters was guided by “Elbow Metaclustering” methodology. It is important to note that the representation presented here corresponds to metaclusters rather than individual clusters. **(D)** The identity of each cluster, color coded in the t-SNE plot, was further visualized using a FlowSOM heatmap. Heatmap shows the frequency of positive expression for surface markers within the indicated T cell populations. Scale bar indicates percent of positive expression from low (blue) to high (red). All frequencies shown were averaged from 10 independent mice.

As shown in **Figure 6A**, Ki67 is mainly expressed by a fraction of CD4+ T cells, that is also CD25^hi^ (south of the t-SNE) and in a lesser extent by a part of CD8+ T cells (north of the t-SNE). The increased Ki67 expression in CD4+ CD25^hi^ population indicates the presence of proliferating Tregs.

The merged t-SNE map was then divided in two different groups based on the treatment. CD4, CD8, activation marker Ki67 and Treg marker CD25 expressions were compared in CD3+ T cells isolated from lymph node of control and treated mice. Slight differences in the frequencies of CD8+ T-cell in treated mice compared to control mice are observed (north of the t-SNE, **Figure 6B**). Moreover, the population of CD4+ CD25^hi^ Ki67+ cells is larger in control mice than in mice treated with the combination (south of the t-SNE, **Figure 6B**).

Further analysis of CD3+ T cells using the FlowSOM algorithm showed 6 metaclusters (**Figure 6C**). Following this, the validation of metaclusters was performed on the t-SNE plot, ensuring the homogeneity of metaclusters. This validation step confirmed the robustness of the identified metaclusters in representing distinct and internally cohesive cell populations within the CD3+ T cells from the lymph node.

The identity of each metacluster color coded in the t-SNE plot was further visualized using a FlowSOM heatmap (**Figure 6D**). For example, Cluster 1, Cluster 5, and Cluster 6 can be readily identified as CD4+ CD25^hi^ Ki67+, CD8+ Ki67+ and CD8+ T cells respectively, by their expression patterns of CD8, CD4, CD25, and Ki67.

### CpG induces a local decrease un the Treg population in the treated tumor

**Figure 7:**
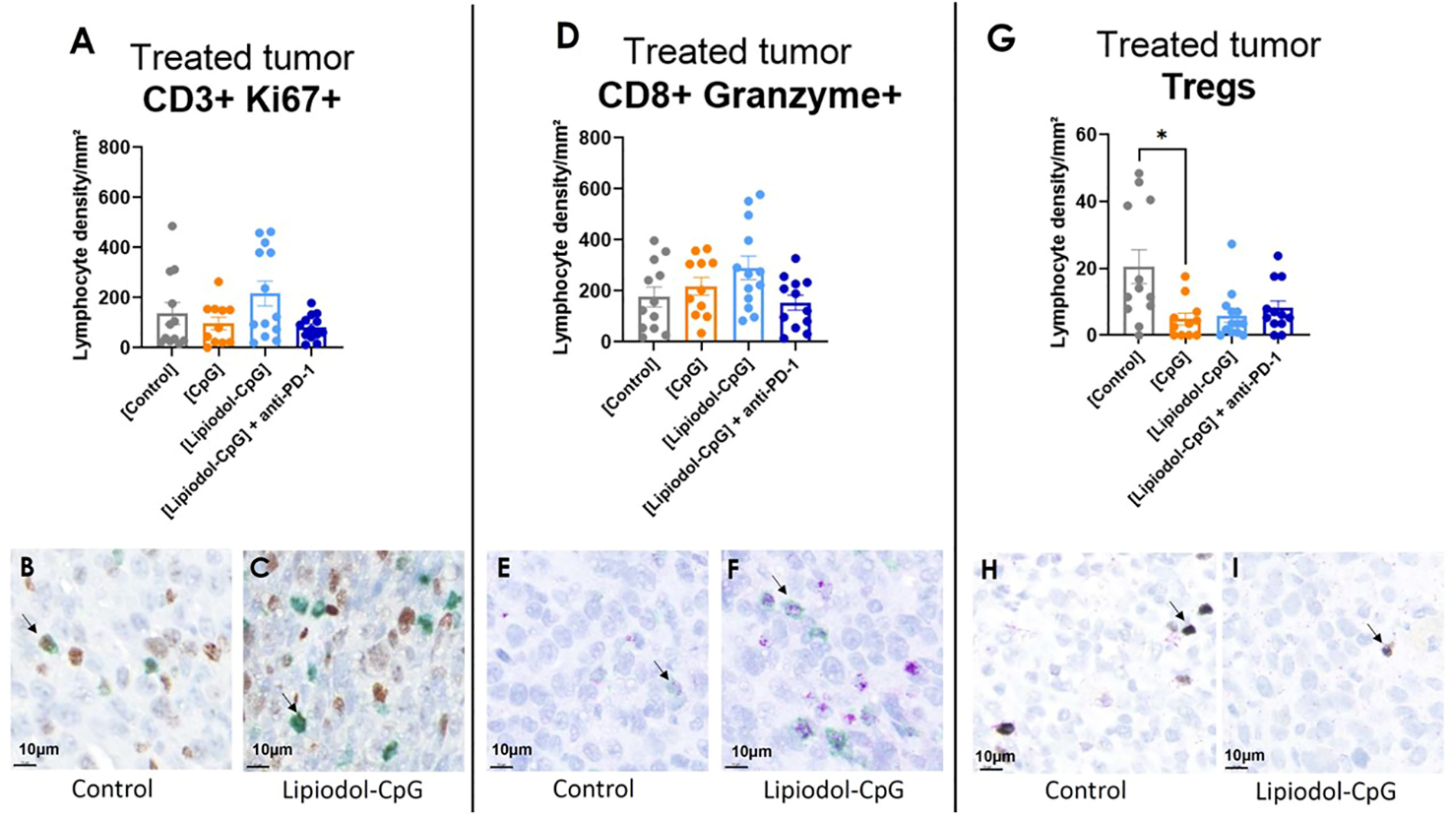
Immunohistochemistry analysis demonstrated a decrease in Tregs infiltration in treated tumors. Lymphocyte density in treated tumor of **(A)** CD3+ Ki67+/mm², **(D)** cytotoxic CD8+ Granzyme + T-cells and **(G)** Treg CD4+ Foxp3+ cells. Double staining was performed. IHC representative images of CD3+ (green) Ki67+ (brown), CD8+ (green) Granzyme+ (purple), and CD4+ (purple) FoxP3+ (silver) cells stained in the main tumor of the control group (**B, E, H** respectively) and of the Lipiodol-CpG group (**C, F, I** respectively). Examples of stained cells are pointed by the black arrows. As Kruskall-Wallis were found positives, Dunn’s post-tests were performed. Data presented as mean ± SEM and individually, * p<0.05.

IHC of treated tumors demonstrated a decreased density of CD4+ FoxP3+ Tregs in all treated groups, which was significant in the CpG group (**Figures 7G–I**). No variation was observed for the proportions of CD3+ Ki67+ and CD8+ Granzyme B+ (**Figures 7A–F**) cells, nor for Pax-5+ B-cells, NCR-1+ NK cells nor PD-1+ cells (data not shown). It is noteworthy that for treated tumors receiving direct i.t. injections, in all mice, and especially in responsive ones, large areas of necrosis impaired IHC analysis.

## Discussion

Lipiodol, used as an intra-arterial drug delivery system for cytotoxic drugs and for immunomodulating molecules (12–14), is approved as an *in situ* therapy for liver malignancies (24, 25). Immunomodulators, such as TLR agonists, are mostly injected locally in preclinical studies (26, 27) and in current clinical trials (NCT03865082, NCT04935229), to activate innate and adaptive immune response in the tumor environment, in addition to avoid systemic toxicity. Anti-PD-1 therapies, on the other hand, are approved for the treatment of advanced cancers and are injected i.v. The aim of the study was to assess the efficacy of a combination of a local treatment with a Lipiodol-immunomodulator emulsion, with systemic injections of anti-PD-1.

This study showed that in a mouse model of CRC, i.t. injection of Lipiodol-CpG emulsion, combined with systemic anti-PD-1, induced an antitumor immune response in injected and distant subcutaneous nodules.

In a first study, a single i.t. injection was performed to select the adjuvant. The Lipiodol-CpG emulsion, especially when combined with systemic anti-PD-1, induced both an increase in pseudo-survival and a decrease in treated tumor growth. No difference was observed between the control group, Lipiodol alone or anti-PD-1 alone in terms of survival and tumor growth.

Lipiodol itself does not have antitumoral properties (28). Besides, the CT26 model, which may be considered as a microsatellite-stable CRC model, has already been characterized as non-responsive to anti-PD-1 monotherapy (29). One of the main mechanisms thought to be the cause of non-responsiveness to PD-1 blockade is that it binds predominantly to immune cells within the TME. The TLR-9 agonist CpG is widely known for its ability to modulate the innate and adaptive immune systems. CpG is an activator of effectors and dendritic cells (30, 31). When emulsified in Lipiodol, it reverses the resistance to anti-PD-1 treatment, presumably by transforming CT26 “cold” tumors into “hot” tumors. Surprisingly, no antitumoral effect of CpG alone was observed, unlike in other published studies on the CT26 model (22, 32), but the latter used multiple injections for a higher total dose of CpG.

Similar to other emulsions used as vaccine adjuvants and known for their immunomodulatory properties (33), using Lipiodol as a carrier may potentiate CpG antitumoral effect thanks to the emulsion formulation. Indeed, Lipiodol-CpG is a stable oil-in-water emulsion that allows longer CpG retention within tumors. Lipiodol, as a drug delivery system, may extend the immune stimulation induced by CpG, leading to a greater antitumoral effect, as it is known for cytotoxic drugs in cTACE. Moreover, since oil-in-water emulsions are more stable with CpG, we can hypothesize that CpG may act as a surfactant, with a similar effect to poly lactic-co-glycolic acid particles (34). However, our Lipiodol-CpG emulsions are direct oil-in-water and not water-in-oil emulsions, recommanded for a cTACE procedure. The water-in-oil emulsions are supposed to improve the delivery of hydrophilic drugs; therefore, our emulsion may not be optimized yet. The stability of Lipiodol-CpG emulsions has been verified only for 24 hours but is not guaranteed beyond 1 day. One limitation of emulsions is that they need to be made extemporaneously. Also, emulsions formulations can, in case a great stability, release only a proportion of the total dose of loaded drug, and not the whole dose (34). Working on the formulation of Lipiodol-CpG emulsions to increase their therapeutic effect is of great interest.

Some studies have suggested a correlation between Lipiodol deposition pattern and treatment response to cTACE in liver malignancies (35, 36). Here, Lipiodol distribution within the tumor was found to be heterogenous, and in responsive mice, Lipiodol was more abundant in the tumor rim. These results may differ from human studies, since Lipiodol emulsions were not injected intra-arterially.

The third step consisted in repeated i.t. injections. This time, CpG alone had a significant and local antitumoral effect. Interestingly, in this model, i) Lipiodol did not modify the effect of CpG, ii) the local antitumoral effect of CpG seemed to be greater than the systemic anti-PD-1 effect, as no difference being observed between the Lipiodol-CpG group and the combination one. We can hypothesize that with this repeated intratumoral administrations protocol, the increased intratumoral retention of the CpG allowed by Lipiodol do not enhance the antitumoral effect during the first eight days post treatment. Moreover, addition of anti-PD-1 did not improve the antitumoral effect of CpG during the first days post treatment.

This design was then completed by injection of a second “metastasis-like” nodule. This provided evidence of an abscopal effect, with a significant slowdown of the untreated nodule growth. To determine whether an enhanced immune response explains the antitumoral effects, the local and systemic immune cell populations were studied following treatment. Among all groups, no difference in all of the studied cells proportions differed between responsive and non-responsive mice, therefore only groups were compared to each other. Therefore, the immune activity was only associated with the global antitumoral response of a group, and not individually with the response within the same group. For all treated mice, spleen weight increased, as already described following CpG treatment (21). Splenomegaly has been correlated with increased MDSC proportions and is associated with poor prognosis in mouse tumor models, and is considered as a biomarker of response to ICI in the clinic (37, 38). The density of CD8+ T-cells increased after treatment within both treated and untreated nodules, especially in the combination group. Although CD8+ proportions were stable in lymphoid organs, more CD8+ Ki67+ proliferating lymphocytes were found in CpG-based treated mice. Several studies have described an expansion of CD8+ proportions following CpG (22, 39) or anti-PD-1 treatments (3). Additionally, the oil-in-water Lipiodol-CpG emulsion may act similarly to squalene-based oil-in-water emulsions, which significantly enhances the CpG-mediated augmentation of CD8+ T-cell responses, contrary to water-in-oil ones (40). Immunohistochemistry of treated tumors did not show any significant variation in granzyme-B+ CD8+ cells among all groups. However, only small areas of the tumors were sampled for staining and may, therefore, not reflect the real density of immune-infiltrating cells.

CpG-ODN, as TLR-9 agonists, are known for their ability to activate B cells and induce their proliferation, especially the B-class CpG (41, 42). Here, no difference in the global B cell proportion in all studied organs have been observed for all treated groups compared to the controls, in both flow cytometry and IHC. It may be explained by the fact that tissues were sampled 8 days following the first i.t. injection, and the delay may not be optimized to observe an effect of B cells. Also, only global B cells have been studied here, it would be interesting to assess subpopulations of B cells following CpG administrations.

IFN-γ, a proinflammatory cytokine released by Th1 T-cells and NK cells, coordinates innate and adaptative immune responses, promotes T-cell differentiation, and is required for CD8+ T-cells to acquire cytotoxic properties (43–45). According to the data reported here, the expression of IFN-γ increased in T-cells from the combination group, as well as its secretion in all treated animals, confirming the CpG-induced systemic T-cell response. The oil-in-water formulation of the emulsion may explain the increase in the CpG induced IFN-γ expression in the Lipiodol-CpG groups, as described by Zhang, et al. (46), who showed higher immune effects with an oil-in-water formulation than water-in-oil in the context of vaccine adjuvants. IFN-γ was reported to drive CpG-induced anemia (47, 48), which may also have occurred in the treated mice, as suggested by the significantly decreased red blood cell counts.

MDSC are immunosuppressive cells that suppress T-cells and their activity and produce anti-inflammatory cytokines (IL-10, TGF-β, etc.), enabling resistance to antitumor immune responses and immunotherapies (23, 49). Surprisingly, flow cytometry analysis revealed a systemic and local increase in MDSC proportions in all treated groups, despite a significant local antitumoral effect. At the same time, no overexpression of IL-10 was observed in treated mice by flow cytometry. These data suggest that an increased proportion of MDSC may not correlate with a more immunosuppressive environment and activity. This is consistent with previously published data demonstrating that TLR-9 activation increases the proportion of MDSC but inhibits their immunosuppressive role in tumor-bearing mice by inducing their maturation and differentiation (32, 50).

In contrast, Tregs were significantly less proliferative in the Lipiodol-CpG and combination groups. Tregs are widely known as immunosuppressive T-cells that inhibit the activation of cytotoxic T-cells and therefore promote tumor proliferation (51). Here, the Lipiodol-CpG emulsion decreased the proportion of proliferative (Ki67+) Tregs without modifying that of Tregs within lymphoid organs, contrary to previous data suggesting that oil-in-water CpG emulsions, more than CpG alone, decrease the proportions of Tregs (40). However, IHC of treated tumors demonstrated a decrease in Tregs in all treated groups, especially in CpG-treated mice. The decrease in the Tregs proportion was also observed, even if not significant, in the lymph node of CpG treated mice only in flow cytometry. This deleterious effect of CpG on Tregs has already been described in a murine model (52). The prognostic impact of Treg cell infiltration in CRC remains controversial. In some studies, high FOXP3+ Tregs infiltration was an unfavorable indicator of patient survival, while other reports reached the opposite conclusion (53, 54). The study reported here demonstrated that antitumoral effects induced by CpG-based treatments are associated with a local decrease in the Tregs population and a systemic decrease in proliferative CD4+ CD25^hi^ cells.

This study has some limitations. First, intra-tumoral injections do not reflect the complexity of an intra-arterial injection followed by vessel embolization as performed in TACE for humans. Also, subcutaneous tumors are not representative of orthotopic tumors, their microenvironment is different, and they do not simulate the spatial context which is as important as the cellular heterogeneity of the TME (55). The “metastatic” nodule was implanted and not spontaneously generated based on a background disease, therefore it does not reflect the gravity of an advanced disease. Moreover, the “metastatic” nodules were too small to perform both immunohistochemistry and flow cytometry. As it had no antitumoral property alone, no investigation of the immune cells populations has been made following an intra-tumoral injection of Lipiodol. No dose titration of CpG has been performed, but only two retreatments. It would have been interesting to modulate the injected dose of CpG, on one hand to optimize the regimen and dosage in this preclinical CT26 model, and on the other hand to evaluate the response of such cancer type to this immunomodulator.

Nevertheless, these data suggest that local treatment with the TLR-9 agonist CpG, emulsified with Lipiodol and combined with systemic anti-PD-1, induces a systemic antitumoral effect. The oil-in-water Lipiodol emulsion allows efficient loading and controlled release of CpG, which induces favorable immune infiltrate modulations especially when combined with anti-PD-1, even in a “metastatic model”. However, the increased immune infiltration of CD8+ cells in tumors, associated with an increase in IFN-γ T-cell expression and secretion, was not associated with a superior antitumoral effect of the emulsion compared to CpG alone in the metachronous metastatic model. Further studies are required to confirm the potential of this combination. Lipiodol, besides its radio-opacity properties, is of great interest in tumor tagging following intra-tumoral or intravascular injections, as it helps in retaining CpG into the tumor. Intra-arterial injection of Lipiodol-based emulsions, currently performed for liver tumor treatment, may lead to more promising results with the full exploitation of Lipiodol properties.

## Data availability statement

The raw data supporting the conclusions of this article will be made available by the authors, upon reasonable request.

## Ethics statement

The animal study was approved by Guerbet ethics committee, N°106. The study was conducted in accordance with the local legislation and institutional requirements. Studies were conducted following the recommendations of the European Convention for the protection of vertebrates Animals use for Experimentation and the local Ethic Committee on Animal care and Experimentation (APAFIS #21714).

## Author contributions

A-LG: Conceptualization, Formal analysis, Investigation, Writing – original draft, Writing – review & editing. NF: Conceptualization, Supervision, Validation, Writing – original draft, Writing – review & editing. MS: Formal analysis, Investigation, Methodology, Writing – review & editing. NB: Investigation, Writing – review & editing. VM: Investigation, Writing – review & editing. RS: Investigation, Writing – review & editing. OB: Investigation, Writing – review & editing. MW: Investigation, Writing – review & editing. CCo: Formal analysis, Writing – review & editing. PR: Conceptualization, Formal analysis, Writing – review & editing. JE: Conceptualization, Investigation, Supervision, Validation, Writing – original draft, Writing – review & editing. CCa: Conceptualization, Formal analysis, Investigation, Supervision, Writing – original draft, Writing – review & editing.
