# Supplementary material for: Antitumoral effect of local injection of TLR-9 agonist emulsified in Lipiodol with systemic anti-PD-1 in a murine model of colorectal carcinoma
Source: Front Immunol. 2024 Jan 16;14:1272246. doi: 10.3389/fimmu.2023.1272246 (PMC10825566; doi:10.3389/fimmu.2023.1272246)
Supplement: Supplementary file 1 [file DataSheet_1.docx]

# Supplemental Material

Supplemental Table 1: Antibodies used for immunohistochemistry analysis

| **Name** | **Manufacturer** | **Clone** | **Host species** | **Dilution** | **HIER** |
| --- | --- | --- | --- | --- | --- |
| CD3 | Cell Signaling | D4V8L | rabbit | 1:20 | CC1 |
| Ki67 | Cell Signaling | D3B5 | rabbit | 1:500 | CC1 |
| CD4 | Cell Signaling | D7D2Z | rabbit | 1:25 | CC1 |
| FoxP3 | Cell Signaling | D608R | rabbit | 1:200 | CC1 |
| Granzyme B | Cell Signaling | E3W4I | rabbit | 1:50 | CC1 |
| CD8 | Abcam | EPR22331-54 | rabbit | 1:500 | CC1 |
| NCR1 | Abcam | EPR23097-35 | rabbit | 1:500 | CC1 |
| Pax5 | Abcam | EPR3730 | rabbit | 1:500 | CC1 |
| CD38 | Abcam | EPR216343 | rabbit | 1:2000 | CC1 |

# Supplemental data

#
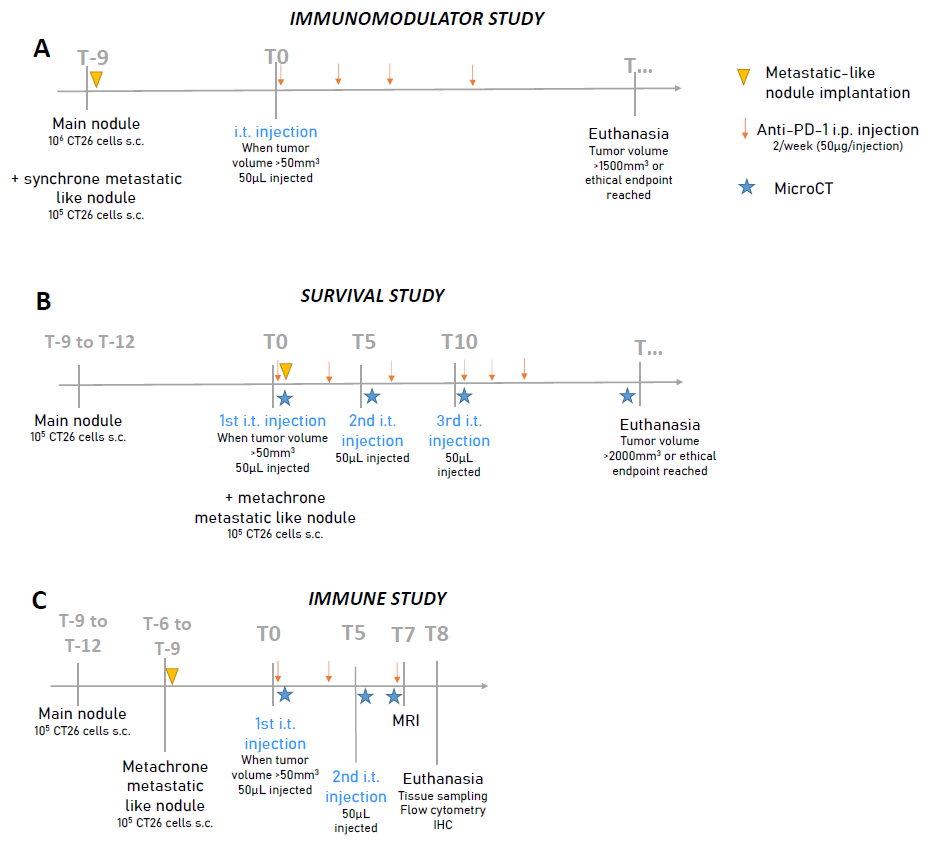


Supplemental Figure 1 : Detailed protocols of the three *in vivo* studies

**Supplemental Table 2: Number of mice euthanized due to their tumor’s ulceration before reaching the maximal volume in the immunomodulator study.**

| Control | 4 |
| --- | --- |
| Anti-PD-1 | 2 |
| Lipiodol | 2 |
| CpG | 1 |
| [Lipiodol-CpG] | 2 |
| [Lipiodol-CpG] + anti-PD-1 | 3 |
| QS21 | 4 |
| [Lipiodol-QS21] | 0 |
| [Lipiodol-QS21] + anti-PD-1 | 0 |
| Poly I:C | 1 |
| [Lipiodol-Poly I:C] | 0 |
| [Lipiodol-Poly I:C] + anti-PD-1 | 1 |


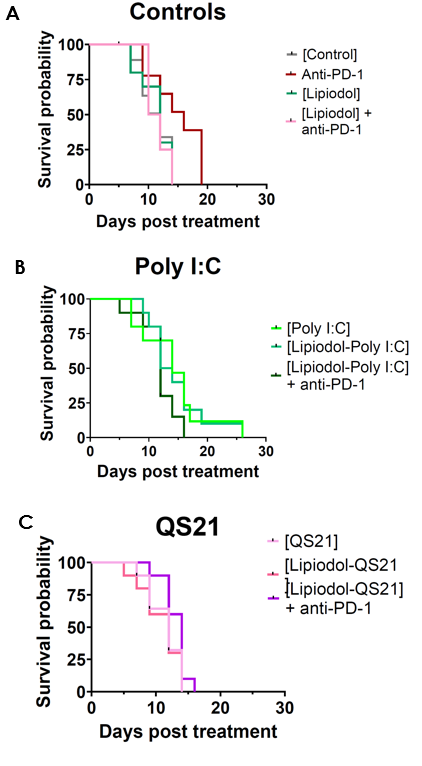


**Supplemental Figure 2: Kaplan-Meyer pseudo-survival curves following intra-tumor injection of (A) control treatments (B) TLR-3 agonist Poly I:C and (C) QS21 (N=10 mice/group).**


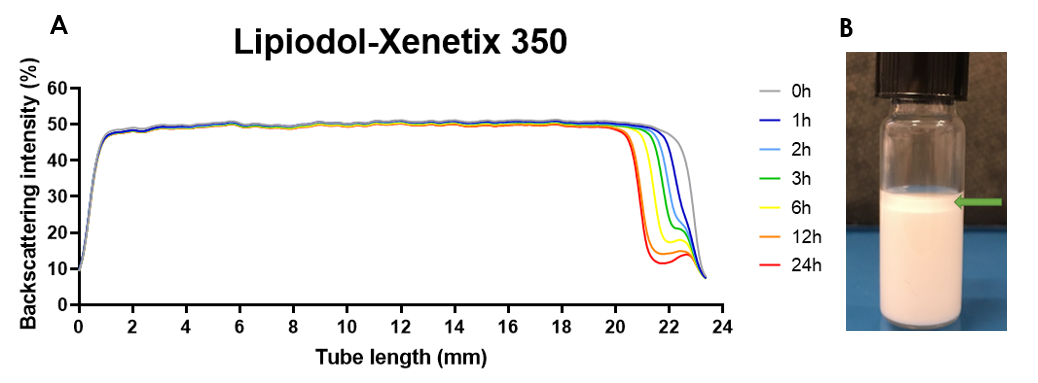


Supplemental Figure 3: Stability of Lipiodol-Xenetix 350 without CpG emulsions.

(A) Turbiscan analysis of a Lipiodol-Xenetix 350 emulsion and (B) Sedimentation observed 24 hours following the formulation process (green arrow).

**
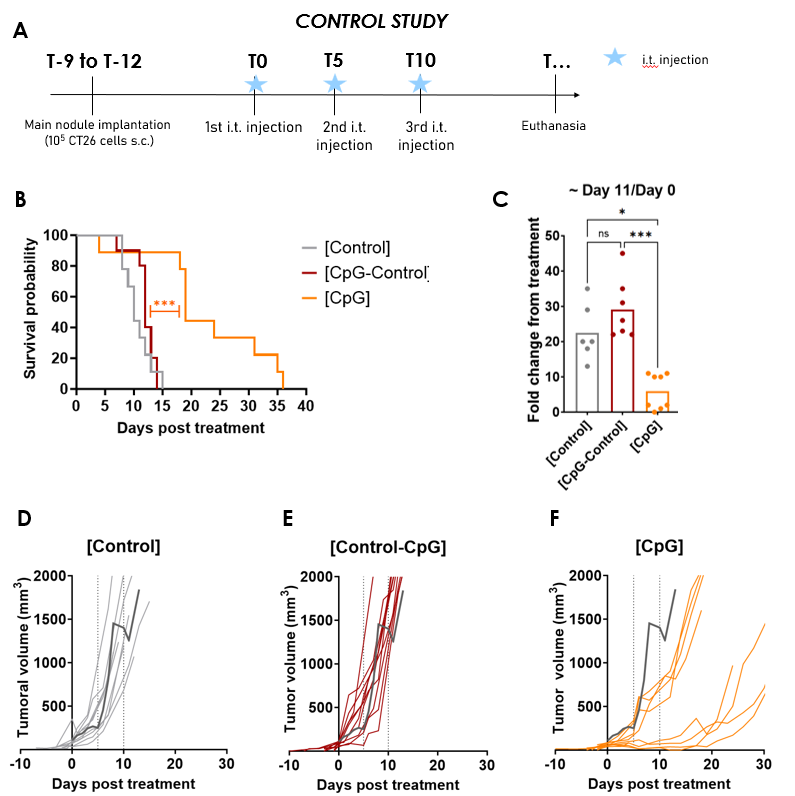
**

**Supplemental Figure 4: CpG-ODN led to a TLR9-dependant anti-tumoral effects on CT26 tumors.**

**(A)** Study design. **(B)** Kaplan-Meyer pseudo-survival curves following the first i.t. injection (N=9-10 mice/group). A log-rank test was performed. **(C)** Fold change in treated-tumor volume at approximatively T11. As Kruskall-Wallis was found positive, a Dunn’s post-test was performed**.** Individual treated-tumor volumes of mice injected with **(D)** control, **(E)** control-CpG, **(F)** CpG intra-tumoral injections. Large grey curves represent the mean of the control group. Data presented as mean and individually, * p<0.05, ** p<0.01, *** p<0.001.


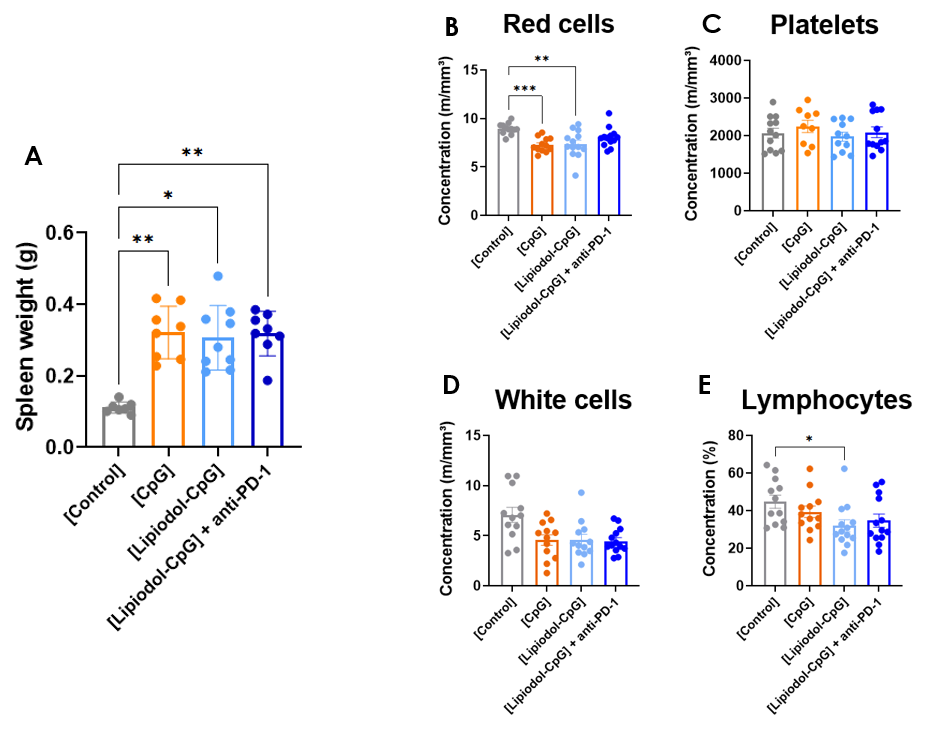


Supplemental Figure 5: Systemic effects of CpG.

**(A)** Spleen were harvested and weighted 8 days following the first i.t. injections. **(B-E)** Blood was sampled at T8, and cell blood count was performed. Data are expressed as mean ± SEM and individually, * p<0.05, ** p<0.01 and *** p<0.001.


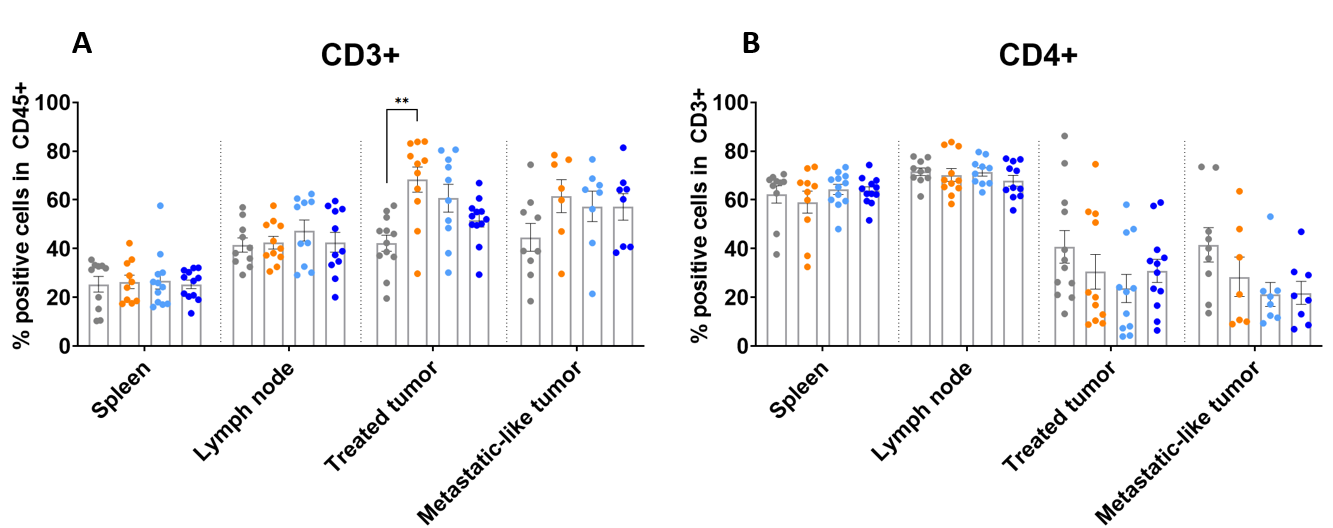
**Supplemental Figure 6: Proportion of (A) CD3+ and (B) CD4+ in the CD45+ subpopulation determined by flow cytometry analysis.**


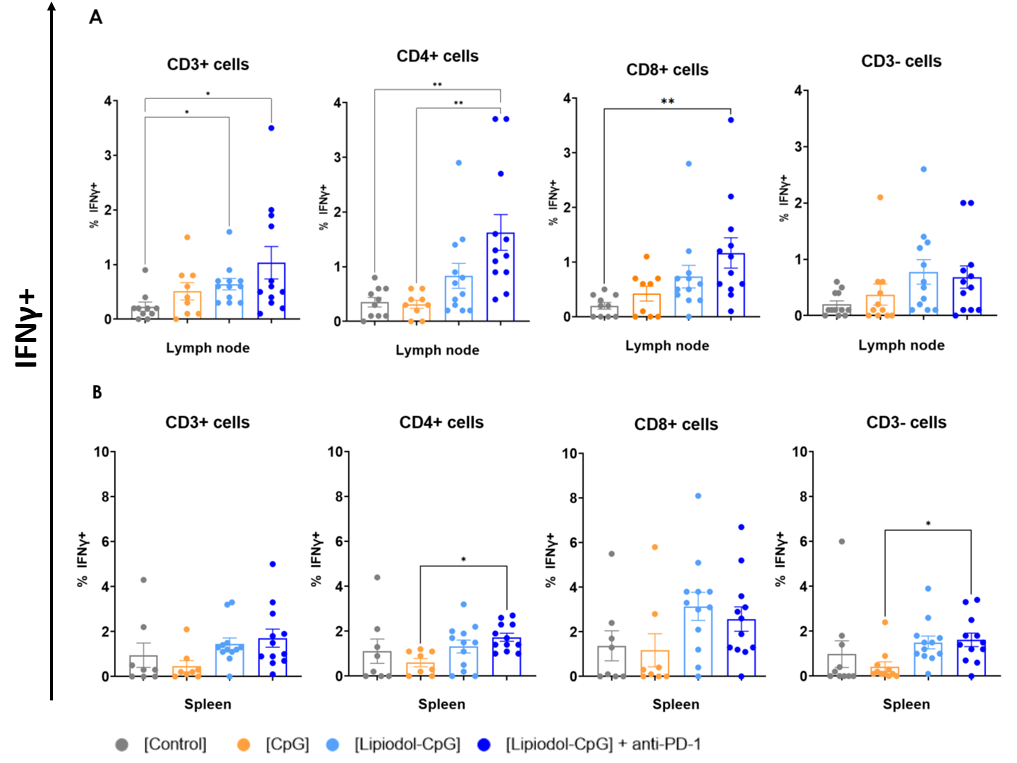


Supplemental Figure 7: IFN-γ secretion by CD3+, CD4+, CD8+ and CD3- producing cells from (A) the lymph node and (B) the spleen.
